# Prevention of carbapenemase-producing Enterobacterales outbreaks: evaluation of a simplified contact screening policy

**DOI:** 10.1016/j.infpip.2026.100597

**Published:** 2026-09-21

**Authors:** Jérémy Picard, Nicolas Galakhoff, Raoul Baron, Geneviève Héry-Arnaud, Clémence Beauruelle, Hervé Le Bars, Séverine Ansart, Gwenaël Cornec, Anne Coste, Aurore Trameçon, Philippe Saliou

**Affiliations:** a Infection Prevention and Control Unit, Brest Teaching Hospital, Brest, France; b Department of Infectious Agents, Pole of Biology-Pathology, University Hospital of Brest Brest, France; c Department of Infectious Diseases, CHU Brest, Brest, France; d Department of Physical and Rehabilitation Medicine, CHU Brest, Brest, France; e Univ Brest, Inserm, EFS, UMR 1078, GGB, Brest, France; f LaTIM, INSERM UMR 1101, Université de Bretagne Occidentale, UBO, Brest, France; g Department of General Practice, Université de Bretagne Occidentale, Brest, France; h ER 7479 SPURBO, Université de Bretagne Occidentale, Brest, France

**Keywords:** carbapenemase-producing Enterobacterales, Contact screening, Targeted screening, Outbreak detection

## Abstract

**Introduction:**

The control of carbapenemase-producing *Enterobacterales* (CPE) relies on screening high-risk patients for carriage, enforcing hygiene measures, and identifying and managing outbreaks of CPE carriers through contact screening. Human and material constraints and the rising prevalence of CPE led to local adaptations in our centre. The main objective was to evaluate the impact of a simplified contact screening policy targeting a limited subset of exposed patients on CPE outbreak detection, and the secondary objective was to identify additional risk factors associated with these outbreaks.

**Methods:**

A retrospective cohort study included hospital stays exceeding 24 h of CPE carriers admitted to medical, surgical, obstetrics, and rehabilitation wards at Brest University Hospital between 2012 and 2022. We used multi-variable mixed-effects logistic regression to assess the impact of our screening policy and identify outbreak risk factors.

**Results:**

A total of 279 hospital stays involving 154 patients were included, with 43 outbreaks identified (15.4%). Three independent factors were associated with outbreak detection: systematic screening of all contact patients (adjusted odds ratio [aOR], 3.65, 95% confidence interval [CI], 1.51–8.84; *P* = 0.004), longer hospital stay (aOR, 1.02 per day, 95% CI, 1.01–1.04; *P* = 0.006), and faecal incontinence of the index patient (aOR, 2.80, 95% CI, 1.10–8.17; *P* = 0.030).

**Discussion:**

Subset contact screening was associated with fewer detected outbreaks than systematic screening, illustrating the trade-off between resource optimization and outbreak detection. As CPE epidemiology evolves, more pragmatic screening strategies focussing on patients or settings at highest risk should be evaluated while maintaining robust infection prevention and control practices.

## Introduction

According to the World Health Organization (WHO), antimicrobial resistance is one of the top ten global threats to public health [1]. The emergence and spread of resistant micro-organisms complicate infection treatment [2], lead to prolonged hospital stays, increase healthcare costs [3], and contribute to higher mortality rates [4].

Carbapenemase-producing Enterobacterales (CPE) are a significant concern due to their role in public health challenges. Efforts to limit their dissemination focus on reducing antibiotic selection pressure and preventing patient-to-patient transmission. European Centre for Disease Prevention and Control (ECDC) recommendations and national recommendations issued by the French High Council for Public Health in 2013, updated in 2019, emphasize the importance of identifying high-risk patients (those hospitalized abroad in the past year), and implementing contact precautions, including single-room isolation and the use of protective clothing. Additional measures include the allocation of dedicated staff to CPE-carrier, if possible, and systematic weekly screening of all individuals exposed to the same medical or paramedical team, i.e. tracking of contact patients [5,6].

Despite their importance, these recommendations are challenging to implement. First, they are resource-intensive due to the human and material constraints faced by healthcare systems. Secondly, a large number of contact patients are lost to follow-up and not screened. The rising prevalence of CPE carriers has further exacerbated these difficulties [7,8].

Epidemic episodes, which are defined by at least one secondary case of CPE-carrying among contact patients, require further control measures [5]. French recommendations for managing epidemic situation include stopping contact-patient transfers between wards, stopping admissions and implementing an organization with different sectors (CPE-carriers, contact-patients, and new patients) and dedicated staff for each sector until the epidemic episode is controlled (with three weeks without any new positive CPE test among contact-patients). These measures are even more expensive and require time for discussion with hospital directors, re-organization of wards and additional staff [9]. Difficulties in implementing these measures can cause adverse effects like a reduction in quality of care and fatigue.

To address these challenges, some hospitals have adapted their strategies, replacing dedicated teams with a ‘forward progression’ approach and implementing targeted contact screening, whereby only a selected subset of patients exposed to CPE carriers, based on assessments by infection prevention and control (IPC) specialists, are screened. [10].

National recommendations for CPE carrier management and outbreak identification were followed until 2017 at our centre. All contact patients of a CPE carrier underwent weekly screening. In 2017, a single, hospital-wide cohorting unit for CPE carriers was established for approximately six months before being discontinued because of difficulties in maintaining continuity of care. In 2018, a simplified CPE management protocol was implemented. This protocol introduced a targeted contact screening strategy, whereby only a selected subset of contact patients, rather than all contacts, underwent weekly screening, without changing the definition of an outbreak. The subset of contact patients was defined by the IPC team according to the duration of exposure to the CPE carrier, the estimated risk of transmission, and geographical proximity. If an outbreak was suspected or identified, screening was immediately extended to all contact patients and additional infection prevention measures, including dedicated healthcare staff, were implemented. Furthermore, in the intensive care units of our centre, all patients were screened for Extended-Spectrum Beta-Lactamase (ESBL)-producing Enterobacterales by rectal swab on admission and weekly thereafter, which occasionally resulted in the incidental detection of CPE carriers.

The main objective of this study was to evaluate the impact of the simplified contact screening strategy on CPE outbreak detection.

The secondary objective was to identify other factors associated with CPE outbreaks detection during the study period.

## Methods

This retrospective cohort study was conducted at Brest University Hospital, France. A carrier patient was defined by the presence of CPE in a rectal swab screening or in any culture sample taken for any diagnostic reason. A carrier patient could be either colonized or infected if they presented clinical signs of infection by the resistant bacteria.

All the CPE samples were identified by the microbiology laboratory of Brest University Hospital, following CA-Recommendations of the Antibiogram Committee of the French Society for Microbiology / European Committee on Antimicrobial Susceptibility Testing (SFM/EUCAST) recommendations for carbapenemase detection [11]. Rectal swabs were explored by culture on ChromID CarbaSmart®. Chek-points carbapenemase-producing organisms polymerase chain reaction (CPO PCR) assays (BDMax™) were used for weekly screening in case of epidemic outbreaks. Enzyme type was identified with Coris Resist-5 OOKNV® or with the help of the National Reference Center for bacterial resistance.

We included hospital stays of more than 24 h for adult patients identified as CPE carriers during the study period spanning from January 1, 2012, to December 31, 2022. Only patients admitted to medical, surgical, obstetric, and rehabilitation wards were included, representing about 700 beds.

An outbreak was defined as the occurrence of at least one secondary case among contact patients involving the same carbapenemase enzyme type while under the care of the same medical or paramedical team.

Hospital stays during which no contact screening was performed were excluded from the analysis, as no epidemic could be detected.

Data were collected from the hospital's IPC team database using the information technology system for carrier-follow-up and laboratory results, and individual patient records, including care plans and discharge summaries.

Information gathered included demographic and clinical characteristics such as age, sex, residence in long-term care facilities, immunosuppression, prior hospitalization within the year, and urinary or faecal incontinence. Clinical details included the patient's CPE status (colonized or infected), the method of detection (diagnostic sampling or systematic screening), microbial species, enzyme resistance type, and known duration of carriage.

The following details of infection control measures were recorded, including hand hygiene compliance score (according to a French health authorities' indicator ‘ICSHA’ [Indicator of Alcohol-Based Hand Rub Consumption] standing for ‘Consumption Index of Hydroalcoholic Solutions’) [12], the most recent local audit scores for infection prevention, prior outbreaks in the same ward within the previous year, and the use and timing of isolation precautions. The screening approach was categorized based on whether all exposed patients (contacts) were screened or a subset only. Calendar year was not included in the multi-variable model because it was strongly correlated with the screening strategy, which changed during the study period and constituted the primary exposure of interest.

Descriptive analyses were conducted to summarize the characteristics of the population and their hospital stays. Statistical tests, including Pearson's chi-squared test or Fisher's exact test for categorical variables, and Student's *t*-test or Mann–Whitney *U* test for continuous variables, were used to compare groups.

Because some patients contributed more than one hospital stay during the study period, standard logistic regression could violate the assumption of independence between observations. Therefore, univariable and multi-variable mixed-effects logistic regression models with a patient-specific random intercept were used to account for repeated hospitalizations. Variables associated with outbreaks with a *P*-value <0.20 in univariable mixed-effects logistic regression were included in a multi-variable mixed-effects logistic regression model with a patient-specific random intercept to account for repeated hospitalizations. The final model was selected using a backward stepwise approach based on likelihood ratio tests. Statistical analyses were performed using RStudio (version 2023.06.1+524).

## Results

A total of 279 hospital stays involving 154 patients carrying CPE were included in the analysis (Figure 1). The characteristics of the study population are detailed in Table I.

**Figure 1:**
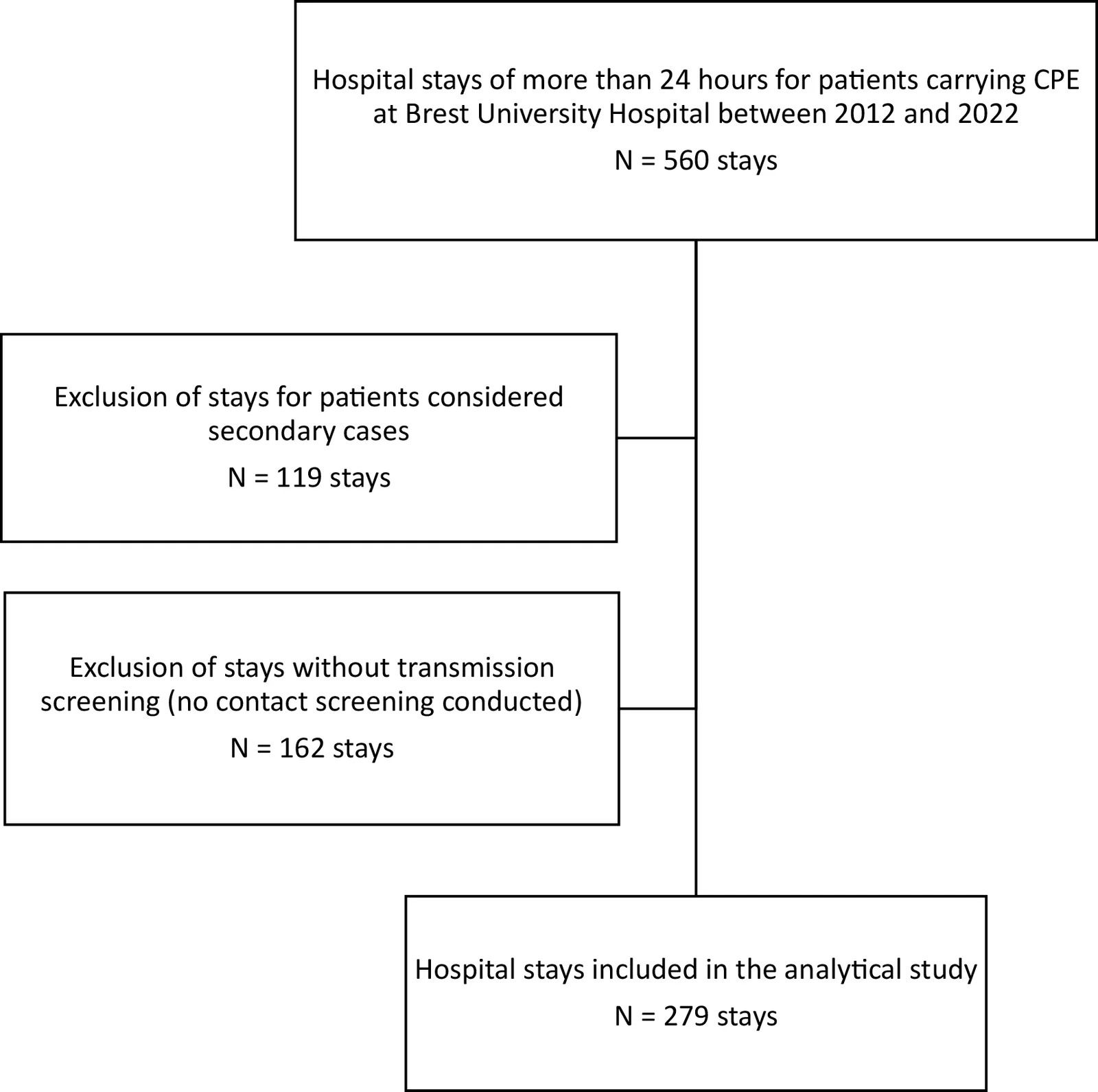
Flow diagram. Carbapenemase-producing Enterobacterales. Hospital stays without contact screening were excluded because outbreak detection was not possible in the absence of contact screening. Some patients contributed more than one hospital stay during the study period.

**Table I:** Characteristics of the 279 hospital stays from 154 CPE carriers

Variable	Total stays (*N* = 279), (%)	Stays without outbreak (*N* = 236), (%)	Stay with outbreak (*N* = 43), (%)
**Male sex**	181 (64.8)	155 (65.6)	26 (60.4)
**Median age at admission (Q1-Q3)**	70.0 (60.5–76.5)	70.0 (61.0–77.0)	68.0 (60.0–75.0)
**Median length of stay (Q1-Q3)**	12.0 (6.5–22.5)	11.0 (6.0–20.25)	17.0 (8.5–41.0)
**Type of ward**			
Medicine	153 (54.8)	130 (55.1)	23 (53.5)
Surgery	45 (16.1)	42 (17.8)	3 (7.0)
Intensive care	56 (20.0)	44 (18.6)	12 (27.9)
Rehabilitation	25 (9.0)	20 (8.5)	5 (11.6)
**Incidental detection**	83 (29.7)	67 (28.4)	16 (37.2)
**Enterobacterales natural resistance group**			
1	59 (21.1)	53 (22.5)	6 (14.0)
2	90 (32.3)	78 (33.1)	12 (27.9)
3	130 (46.6)	105 (44.5)	25 (58.1)
**CPE infection**	61 (21.9)	49 (20.8)	12 (27.9)
**Median known carrier duration (days)**	42.0 (14.0–172.5)	47.0 (14.0–191.0)	40.0 (13.5–90.0)
**Long-term care facility residence**	16 (5.7)	12 (5.1)	4 (9.3)
**Recent antibiotherapy (<1 month)**	179 (64.2)	147 (62.3)	32 (74.4)
**Urinary incontinence**	231 (82.8)	193 (81.8)	38 (88.4)
**Fecal incontinence**	205 (73.5)	167 (70.8)	38 (88.4)
**Central veinous catheter**	88 (31.5)	68 (28.8)	20 (46.5)
**Hygiene assistance**	225 (80.6)	183 (77.5)	37 (86.0)
**Immunosuppression**	105 (37.6)	89 (37.7)	16 (37.2)
**Contact precautions at admission**	192 (68.8)	167 (70.8)	25 (58.1)
**Single room at admission**	263 (94.3)	224 (94.9)	39 (90.7)
**Ward hand hygiene score**			
A	147 (52.7)	122 (51.7)	25 (58.1)
B	59 (21.1)	49 (20.8)	10 (23.3)
C	73 (26.2)	65 (27.5)	8 (18.6)
**Local audit score for infection prevention (Q1-Q3) (out of 100)**	65 (53–71)	65 (53–71)	60 (53–69)
**Ward recent history of outbreak (<1 year)**	132 (47.3)	113 (47.9)	19 (44.2)
**Screening of all contacts**	48 (17.2)	34 (14.4)	14 (32.6)
**Mean number of screenings per week (** ± **SD)**	8.2 ± 24.4	5.1 ± 11.8	25.06 ± 53.1

Enterobacterales groups: Classification based on the intrinsic β-lactam resistance phenotype of Enterobacterales. Group 1: naturally susceptible species (e.g. Escherichia coli and Proteus mirabilis); Group 2: species producing a constitutive chromosomal penicillinase (e.g. Klebsiella pneumoniae, Klebsiella oxytoca, and Citrobacter koseri); Group 3: species producing an inducible chromosomal AmpC cephalosporinase (e.g. Enterobacter cloacae complex, Klebsiella aerogenes, Citrobacter freundii, Serratia marcescens, Morganella morganii, and Providencia spp.).

Ward hand hygiene score: French ICSHA (Indicator of Alcohol-Based Hand Rub Consumption), calculated as the annual ratio of actual alcohol-based hand rub consumption to the expected consumption, expressed as a percentage. Categories: A (≥100%), B (80–99%), and C (<80%). CPE, carbapenemase-producing Enterobacterales.

OXA-48-like carbapenemases accounted for 90.6% of isolates, followed by New Delhi metallo-β-lactamase (NDM) (6.4%), Klebsiella pneumoniae carbapenemase (KPC) (1.7%), and IMI (0.4%). Carbapenemase type could not be retrospectively determined for nine hospital stays. The most frequent bacterial species were *Enterobacter cloacae* complex (28.4%) followed by *Citrobacter freundii* (21.0%), *Escherichia coli* (18.9%) and *Klebsiella pneumoniae* (14.6%). CPE infection was documented in 17.9% of carriers.

During these hospital stays, 43 outbreaks were identified, corresponding to 15.4% of included stays.

In univariable analyses (Table II), longer hospital stay (odds ratio [OR], 1.02 per additional day, 95% confidence interval [CI], 1.01–1.03, *P* = 0.0047), shorter known duration of CPE carriage (OR, 0.997 per day, 95% CI, 0.99–1.00, *P* = 0.0048), fecal incontinence (OR, 3.14, 95% CI, 1.19-8.31, *P* = 0.021), the presence of a central venous catheter (OR, 2.14, 95% CI, 1.11–4.17, *P* = 0.024), and systematic screening of all contact patients compared with subset screening (OR, 2.91, 95% CI, 1.31–6.43, *P* = 0.0084) were associated with outbreak detection.

**Table II:** Mixed-effects logistic regression analyses of factors associated with CPE outbreak detection

Variable	Univariable mixed-effects model		Multi-variable mixed-effects model	
	OR [CI 95%]	*P*-value	aOR [CI 95%]	*P*-value
**Male sex**	0.80 [0.40; 1.60]	*P* = 0.52	-	
**Age at admission (per year)**	0.99 [0.97; 1.02]	*P* = 0.67	-	
**Length of stay**	1.02 [1.01; 1.03]	*P* = 0.0047∗	1.02 [1.01; 1.04]	0.006 ∗
**Type of ward**			-	
Medicine	réf.			
Surgery	0.41 [0.11; 1.41]	*P* = 0.16		
Intensive care	1.54 [0.71; 3.35]	*P* = 0.27		
Rehabilitation	1.41 [0.48; 4.14]	*P* = 0.53		
**Incidental detection**	1.49 [0.76; 2.95]	*P* = 0.25	-	
**Enterobacterales natural resistance group**			-	
1	réf.			
2	1.36 [0.48; 4.14]	*P* = 0.56		
3	2.10 [0.81; 5.44]	*P* = 0.13		
**CPE infection**	1.48 [0.70; 3.09]	*P* = 0.30	-	
**Known carrier duration (days)**	0.997 [0.99; 1.00]	*P* = 0.0048∗	-	
**Long-term care facility residence**	1.91 [0.59; 6.24]	*P* = 0.28	-	
**Recent antibiotherapy (<1 month)**	1.76 [0.85; 3.67]	*P* = 0.13	-	
**Urinary incontinence**	1.69 [0.63; 4.57]	*P* = 0.30	-	
**Fecal incontinence**	3.14 [1.19; 8.31]	*P* = 0.021∗	2.80 [1.10; 8.17]	0.03 ∗
**Central veinous catheter**	2.14 [1.11; 4.17]	*P* = 0.024∗	-	
**Hygiene assistance**	1.76 [0.69; 4.49]	*P* = 0.24	-	
**Immunosuppression**	0.98 [0.50; 1.93]	*P* = 0.95	-	
**Contact precautions at admission**	0.57 [0.29; 1.12]	*P* = 0.10	-	
**Single room at admission**	0.52 [0.16; 1.72]	*P* = 0.29	-	
**Ward hand hygiene score**			-	
A	réf.			
B	1.00 [0.45; 2.23]	*P* = 0.99		
C	0.60 [0.26; 1.41]	*P* = 0.24		
**Local audit score for infection prevention**	0.99 [0.96; 1.02]	*P* = 0.47	-	
**Ward recent history of outbreak (<1 year)**	0.86 [0.42; 1.74]	*P* = 0.68	-	
**Screening of all contacts**	2.91 [1.31; 6.43]	*P* = 0.0084∗	3.65 [1.51; 8.84]	0.004 ∗

aOR, adjusted odds ratio; CI, confidence interval; CPE, carbapenemase producing Enterobacterales; OR, odds ratio.

Enterobacterales groups: Classification based on the intrinsic β-lactam resistance phenotype of Enterobacterales. Group 1: naturally susceptible species (e.g. Escherichia coli, Proteus mirabilis); Group 2: species producing a constitutive chromosomal penicillinase (e.g. Klebsiella pneumoniae, Klebsiella oxytoca, and Citrobacter koseri); Group 3: species producing an inducible chromosomal AmpC cephalosporinase (e.g. Enterobacter cloacae complex, Klebsiella aerogenes, Citrobacter freundii, Serratia marcescens, Morganella morganii, and Providencia spp.).

Ward hand hygiene score: French ICSHA (Indicator of Alcohol-Based Hand Rub Consumption), calculated as the annual ratio of actual alcohol-based hand rub consumption to the expected consumption, expressed as a percentage. Categories: A (≥100%), B (80–99%), and C (<80%).

∗ Statistically significant at p < 0.05.

The final mixed-effects multi-variable model identified three independent factors associated with outbreak detection: longer hospitalization (adjusted OR [aOR], 1.02, 95% CI, 1.01–1.04, *P* = 0.006), faecal incontinence in the index patient (aOR, 2.80, 95% CI, 1.10–8.17, *P* = 0.03), and systematic screening of all contact patients as compared with subset screening (aOR, 3.65, 95% CI, 1.51–8.84, *P* = 0.004).

## Discussion

This single-centre study, conducted over more than a decade, identified three independent factors associated with outbreak detection involving CPE following the hospitalization of a colonized patient. These factors include the systematic screening of all patient contacts, the duration of hospital stay of the colonized patient, and faecal incontinence.

The systematic screening of all patient contacts was associated with a higher frequency of outbreak detection. This finding was expected, as screening all contact patients increases the probability of identifying at least one secondary CPE carrier and therefore detecting an outbreak. Although the targeted screening strategy relied on an IPC risk assessment to select the contact patients considered most likely to have been exposed, reducing the number of screened contacts inevitably decreases the likelihood of detecting every transmission event. As simplified screening strategies become increasingly implemented to optimize resources, this trade-off should be recognized when interpreting outbreak detection rates. In late 2017, our centre introduced a local policy of subset screening to optimize resources, whereby only a selected sample of contact patients, rather than all contacts, underwent weekly screening. Prior to this change, all contact patients were systematically screened weekly, alongside infection prevention measures such as contact precautions and patient cohorting. The shift to a targeted screening approach was motivated by several challenges, including the logistical burden of universal screening and its associated costs, especially in cases where colonized patients were distributed across multiple units. Beyond its effect on the measured outcome, failure to identify secondary carriers could delay the recognition of transmission and the implementation of additional control measures, potentially allowing further spread. In our centre, suspected or identified outbreaks prompted immediate extension of screening to all contact patients and reinforcement of infection prevention measures, including dedicated healthcare staff. These measures were intended to mitigate this risk, but their activation depended on transmission being suspected or detected; they therefore cannot exclude transmission remaining unrecognized. In the most recent ‘Evaluation of measures to prevent the transmission of emerging highly resistant bacteria (EVA-BHRe)’ audit conducted by a French Centre for Support and Prevention of Healthcare-Associated Infections in 2020 and 2021 [10], 89.7% of participating services reported adhering to weekly contact screening recommendations. At the national level, Santé Publique France observed a simultaneous rise in reported cases and a decline in the implementation of complementary contact precautions at admission (33% in 2022 vs 40% in 2019), specific measures following case identification (45% of forward progression or dedicated paramedical staff vs 68% in 2019), and contact screenings around index cases (79% of cases only) [13]. In 2020, another centre proposed this modification for a known carrier placed under contact precautions upon admission, in a ward where transmission prevention is well controlled (assessed by hand hygiene compliance, excreta management, and experience with CPE carriers), weekly screening of a subset of contacts would be performed [14]. This strategy could represent a pragmatic way to address the challenge of CPE control among the many competing priorities in PC.

The duration of hospitalization of colonized patients was also associated with outbreaks, consistent with findings in the literature. Longer stays increase the exposure time for healthcare-associated transmission to occur [15,16]. This may be compounded by difficulties in maintaining consistent adherence to contact precautions over prolonged periods.

Faecal incontinence was another significant independent factor for transmission events. Defined as a patient's inability to use the toilet independently, faecal incontinence likely facilitates cross-transmission through both direct handborne transfer and environmental contamination. Given that the primary reservoir for CPE is the gastrointestinal tract, these findings align with previous studies emphasizing the role of faecal management in preventing environmental dissemination. This aligns with the findings of Lerner *et al.* [17], who demonstrated that faecal continence was a protective factor against environmental contamination in patients colonized with KPC-producing CPE and which have subsequently been supported by recent reviews on CPE transmission dynamics [18]. However, this factor was not identified in multi-variate analyses in two retrospective French studies [15,19]. Nevertheless, the confidence interval around our estimate was relatively wide, indicating limited precision in the magnitude of the association, likely due to the limited number of outbreak events. Therefore, this finding should be interpreted with caution and confirmed in larger studies. A 2017 update of standard precautions by the French Society for Hospital Hygiene emphasized key measures, including appropriate use of personal protective equipment, rigorous hand hygiene, and the exclusive use of disposable containers for managing excreta [20]. Reinforcing these measures at the institutional level could further reduce the risk of cross-transmission.

Interestingly, no significant association was found between an identified outbreak and the fortuitous discovery of CPE carriage during diagnostic or routine multi-drug-resistant organism (MDRO) screening. While such discoveries logically delay the implementation of contact precautions, their impact on outbreak detection appeared limited in this study. Similarly, adherence to hand hygiene and infection control scores, assessed annually or biennially, did not show significant associations with outbreak detection. These metrics may not adequately capture real-time variations in infection control practices during hospitalization [15,18,21].

Recent antibiotic consumption by the index patient was not found to be a significant factor in transmission events. Previous research has shown that antibiotic exposure, both before and during hospitalization, is associated with secondary acquisition of CPE among contact patients [15]. In our study, the unit of analysis was the hospitalization of the index CPE carrier; although contact patients were used to identify outbreak events, their individual characteristics and antibiotic exposure were not analysed and therefore could not be evaluated as potential risk factors.

This study has several limitations. First, even if it included all available CPE carrier cases over the study period, statistical power may have been insufficient to identify additional risk factors. Many admissions were excluded because no contact screening was performed. These were predominantly very short hospital stays (<48 h), during which contact screening was often not feasible. Indeed, identifying contact patients and co-ordinating specimen collection throughout the hospital ward requires time and organizational resources that are generally unavailable during short admissions. Consequently, some degree of selection bias cannot be excluded, and our findings may not be fully generalizable to all CPE carrier admissions, particularly those involving very short hospitalizations. However, because the primary outcome relied on the identification of secondary cases through contact screening, admissions without screening could not be reliably classified with respect to outbreak occurrence and were therefore not suitable for analysis. The available data did not provide an assessment of transmission independent of the contact screening strategy. We therefore could not quantify the number of secondary carriers or outbreaks that may have been missed, or determine whether unrecognized transmission persisted during the observation period.

Furthermore, outbreaks were defined as the occurrence of at least one contact patient carrying a CPE producing the same carbapenemase enzyme as the index patient. However, molecular typing or whole-genome sequencing was not systematically performed and therefore direct transmission between patients could not be microbiologically confirmed. Consequently, some events classified as outbreaks may have represented independent acquisitions of genetically unrelated strains producing the same carbapenemase enzyme, leading to a possible overestimation of outbreak occurrence. Although the prevalence of community-acquired CPE in our region is unknown, it is likely to be non-negligible based on routine multi-drug-resistant organism screening performed on admission to intensive care units. Then, the study excluded cases where cohorting was implemented, as this intervention often precludes contact screening. Although cohorting is a recommended infection control strategy, its cost-effectiveness remains debated, particularly in the context of staffing shortages in healthcare settings [[7], [8], [9],^15^,^22^,^23^].

Additionally, colonization pressure within hospital units was not accounted for in this study. The simultaneous presence of multiple colonized patients in the same unit may increase the risk of CPE transmission. At the individual level, incomplete data on digestive colonization density precluded its inclusion as a risk factor. This is an important limitation, as colonization density is known to be variable and may correlate with environmental contamination risk [17]. Similarly, the study did not examine antibiotic use at the ward or contact-patient level, despite robust evidence linking broad-spectrum antibiotic pressure to both transmission and acquisition of multi-drug-resistant organisms [18,21]. This underscores the need to continue strengthening antimicrobial stewardship policies at both institutional and regional levels.

Our findings should be interpreted within the broader context of CPE control. Current French recommendations apply the same infection prevention measures to all hospitalized CPE carriers, regardless of their infectious status, reflecting the need to prevent transmission in healthcare settings. As CPE epidemiology continues to evolve and colonization pressure increases over time, optimizing the balance between resource use and outbreak detection will remain an important challenge. In this context, targeted contact screening may represent a pragmatic approach in selected settings, provided that it is integrated into a comprehensive IPC strategy and accompanied by careful risk assessment. However, our findings do not establish the safety of reducing contact screening. Prospective multi-centre evaluations incorporating systematic follow-up of exposed patients and assessment of subsequent transmission are needed before broader implementation can be supported.

In, conclusion, this study provides insight into the impact of a targeted contact screening strategy on the detection of CPE outbreaks in a large tertiary-care hospital. Compared with systematic screening of all contact patients, targeted screening was associated with a lower probability of detecting outbreak events, highlighting the trade-off between resource optimization and outbreak detection.

Future research should focus on optimizing outbreak detection while limiting the burden associated with universal screening. Digital tools for patient tracking, automated alerts, and infection prevention surveillance may help improve the efficiency of contact investigations. In addition, advances in molecular epidemiology could provide a better understanding of transmission dynamics and support more targeted infection prevention strategies. Effective collaboration between IPC teams, microbiology laboratories, and infectious disease specialists will remain essential to adapt CPE control strategies to evolving epidemiological and healthcare resource constraints.

## CRediT authorship contribution statement

**Jeremy Picard:** Writing – review & editing, Writing – original draft, Validation, Methodology, Investigation, Formal analysis, Data curation, Conceptualization. **Nicolas Galakhoff:** Writing – review & editing, Investigation. **Raoul Baron:** Writing – review & editing, Conceptualization. **Geneviève Héry-Arnaud:** Writing – review & editing, Resources. **Clémence Beauruelle:** Writing – review & editing, Investigation. **Hervé Le Bars:** Writing – review & editing, Investigation. **Séverine Ansart:** Writing – review & editing, Validation, Supervision, Methodology. **Gwenaël Cornec:** Writing – review & editing, Methodology. **Anne Coste:** Writing – review & editing, Supervision, Methodology. **Aurore Trameçon:** Writing – review & editing, Validation, Methodology, Investigation. **Philippe Saliou:** Writing – review & editing, Writing – original draft, Validation, Supervision, Methodology, Investigation, Formal analysis, Conceptualization.

## Ethical approval

This study was approved by the Ethics Committee of Brest Hospital (B2025CE.08).

## Declaration of generative AI and AI-assisted technologies in the manuscript preparation process

During the preparation of this work, the authors used ChatGPT (OpenAI) to assist with language editing and refinement of the manuscript. The authors reviewed and edited the output as needed and take full responsibility for the content of the published article.

## Funding sources

None.

## Conflict of interest statement

None declared.
